# Bacterial and fungal communities in sub-Arctic tundra heaths are shaped by contrasting snow accumulation and nutrient availability

**DOI:** 10.1093/femsec/fiae036

**Published:** 2024-03-28

**Authors:** Minna K Männistö, Saija H K Ahonen, Lars Ganzert, Marja Tiirola, Sari Stark, Max M Häggblom

**Affiliations:** Natural Resources Institute Finland, Ounasjoentie 6, FI-96200 Rovaniemi, Finland; Ecology and Genetics Research Unit, University of Oulu, Pentti Kaiteran katu 1, FI-90014 Oulu, Finland; Natural Resources Institute Finland, Ounasjoentie 6, FI-96200 Rovaniemi, Finland; Plankton and Microbial Ecology, Leibniz Institute of Freshwater Ecology and Inland Fisheries, Zur alten Fischerhütte 2, 16775 Stechlin, Germany; Department of Biological and Environmental Science, University of Jyväskylä, Survontie 9, FI-40014 Jyväskylä, Finland; Arctic Centre, University of Lapland, Pohjoisranta 4, Fl-96101 Rovaniemi, Finland; Natural Resources Institute Finland, Ounasjoentie 6, FI-96200 Rovaniemi, Finland; Department of Biochemistry and Microbiology, Rutgers University, 76 Lipman Drive, New Brunswick, NJ 08901, United States

**Keywords:** bacterial community, climate change, fungal community, snow, tundra, winter

## Abstract

Climate change is affecting winter snow conditions significantly in northern ecosystems but the effects of the changing conditions for soil microbial communities are not well-understood. We utilized naturally occurring differences in snow accumulation to understand how the wintertime subnivean conditions shape bacterial and fungal communities in dwarf shrub-dominated sub-Arctic Fennoscandian tundra sampled in mid-winter, early, and late growing season. Phospholipid fatty acid (PLFA) and quantitative PCR analyses indicated that fungal abundance was higher in windswept tundra heaths with low snow accumulation and lower nutrient availability. This was associated with clear differences in the microbial community structure throughout the season. Members of *Clavaria* spp. and *Sebacinales* were especially dominant in the windswept heaths. Bacterial biomass proxies were higher in the snow-accumulating tundra heaths in the late growing season but there were only minor differences in the biomass or community structure in winter. Bacterial communities were dominated by members of Alphaproteobacteria, Actinomycetota, and Acidobacteriota and were less affected by the snow conditions than the fungal communities. The results suggest that small-scale spatial patterns in snow accumulation leading to a mosaic of differing tundra heath vegetation shapes bacterial and fungal communities as well as soil carbon and nutrient availability.

## Teaser

Snow cover conditions affect fungal and bacterial biomass and community structure in sub-Arctic tundra heaths.

## Introduction

High-latitude soils store approximately half of the global soil organic carbon, and hence it is of major concern how global climate change will affect decomposition rates and C flux to the atmosphere in these regions (Tarnocai et al. 2009, Schuur et al. 2015). Arctic regions are warming up to four times faster than the global average (Rantanen et al. 2022), with the cold season months warming at a much faster rate than the growing season (Mikkonen et al. 2015, Rantanen et al. 2022). In northern Fennoscandia, in addition to increasing winter month temperatures, the number of frost days has declined, exceptionally cold winter days have decreased, while exceptionally warm days and the number of freeze–thaw cycles have increased (Mikkonen et al. 2015, Kivinen et al. 2017, Lepy and Pasanen 2017). Precipitation is also increasing in high-latitude ecosystems (Groisman et al. 2005, McCrystall et al. 2021), which influences the amount of snow that accumulates during winter. The depth and insulating properties of the snowpack, however, depend on the form of precipitation. As the extremely warm winter days are increasing in Arctic ecosystems it is predicted that the precipitation will be more and more in the form of rain instead of snow, and the thickness and timing of snowpack may be reduced (Bintanja and Andry 2017, McCrystall et al. 2021). Furthermore, increased winter thaw and rain on snow events diminish the insulating properties of snow (Serreze et al. 2021) and may lead to colder soil temperatures during winter (Groffman et al. 2001). In addition to direct changes in precipitation and soil temperature, climate warming is altering wintertime soil temperature regimes also indirectly through changes in the dominant vegetation. Tundra ecosystems are undergoing strong expansion and increase of shrubs (Sturm et al. 2001b), which may control the wintertime soil temperatures by trapping more snow that leads to increased insulation (Sturm et al. 2001a). Due to the complex ways by which climate change is altering snow cover, there is a high need to better understand the role of snow cover for soil microbial communities.

The wintertime microbial activity contributes to a significant part of annual carbon cycling in tundra ecosystems (Oechel et al. 1997, Fahnestock et al. 1999, Euskirchen 2012 et al. 2012, Natali et al. 2019), and in boreal and Arctic soils overwinter CO_2_ effluxes may even exceed plant carbon uptake during the growing season (Natali et al. 2019). Microbial activity and CO_2_ flux continue in tundra soils through the winter down to −20°C (Natali et al. 2019). In Fennoscandian tundra soil, bacterial activity down to −16°C was identified with stable isotope probing with distinct communities growing at different subzero temperatures (Gadkari et al. 2019). Around and below 0°C, soil microbial activities become, however, increasingly temperature sensitive (Mikan et al. 2002, Sullivan et al. 2008, Tilston et al. 2010), and microbial substrate utilization may shift to decomposition of more labile organic matter (OM) such as fresh litter and root exudates or microbial biomass and by-products further affecting the C and nutrient cycling (Mikan et al. 2002, Schimel et al. 2004, Grogan and Jonasson 2005, Sturm et al. 2005). Under a thick snow cover, soil temperatures are decoupled from air temperatures and may remain close to 0°C throughout the coldest months (Schimel et al. 2004, Männistö et al. 2013, Pattison and Welker 2014, Convey et al. 2018, Way and Lewkowicz 2018, Rixen et al. 2022). Snow cover conditions thus control cold season microbial activities and deeper snow may enhance winter microbial respiration even to the degree that it switches the ecosystem annual net carbon exchange from a sink to source (Nobrega and Grogan 2007, Natali et al. 2019). However, increased cold season microbial respiration may have legacy effects to the following growing seasons. Snow manipulation experiments have indicated that increased microbial activity during winter may lead to depletion of labile C and over time to reduced growing season CO_2_ emissions (Semenchuk et al. 2016, Sullivan et al. 2020). Moreover, increased nitrogen (N) mineralization under deeper snow cover and associated higher soil temperatures may have significant consequences for plant growth and consequently carbon cycling especially in the nutrient limited tundra ecosystems (Schimel et al. 2004)

The current understanding of the effects of snow depth on soil microbial communities mainly derives from experimental manipulation of the snowpack. In subalpine grassland and temperate deciduous forest soils, strong changes were reported in the bacterial and fungal communities under reduced snow cover during winter, but these differences leveled out during the growing season (Aanderud et al. 2013, Gavazov et al. 2017). In boreal forest soil, no effect of snow depth or snow properties was detected in bacterial or fungal communities either before or after spring thaw or in the late growing season (Männistö et al. 2018). On the other hand, strong shifts were reported in microbial communities of alpine grasslands during spring thaw and these were linked to shifts in microbial functions and biogeochemical fluxes suggesting that changes in the timing of spring thaw may have important consequences for the ecosystem functioning (Broadbent et al. 2021). In acidic tundra soils, bacterial community structure was significantly affected by snow depth, which was associated with changes in edaphic factors (Ricketts et al. 2016). Semenova et al. (2016) reported changes in the abundance and community structure of saprotrophic, ectomycorrhizal, plant pathogenic, and lichen- and bryophyte-associated fungal guilds with deeper snow. These changes were not entirely associated with shifts in vegetation but there were indications that fungal communities in the Arctic may exhibit faster turnover which is influenced e.g. by soil nutrient availability and dynamics of other microbial groups. Experimental manipulations of snow cover conditions have shown both differing vegetation (Olofsson et al. 2009) and higher soil microbial N and bacterial counts (Buckeridge and Grogan 2010) with deepened snow. Together, these studies indicate that there may be large differences in the resilience and sensitivity of bacterial and fungal communities to changes in winter soil temperature and moisture in different soil ecosystems. In addition, experimental manipulations of snow depth are often of short duration, and may thus not necessarily depict long-term changes that would be mediated by the combination of differing vegetation, nutrient availability, and temperature regimes, which all change in response to differing snow cover.

In tundra landscapes, the small-scale variation in topography, which in turn affects wintertime snow accumulation, leads to a mosaic of habitats with differing snow conditions and dominant vegetation over a gradient from low and absent snow cover to snow-accumulating (SA) sites (Oksanen and Virtanen 1995, Niittynen et al. 2020). Exposed ridges that are windswept (WS) remain nearly snow-free throughout the winter, while sheltered slopes and depressions accumulate snow already early in winter that remains until early summer. The WS and SA habitats exhibit widely divergent wintertime soil temperatures and the duration of the snow-free period, which gives rise to divergent plant coverage, amount of litter, and nutrient availability (Niittynen et al. 2020). In Fennoscandian tundra, *Empetrum nigrum* ssp. *hermaphroditum* heaths dominate the WS heaths while communities rich in *Vaccinium myrtillus* L. occur as strands between *Empetrum* heaths and grass-dominating snow-beds (Oksanen and Virtanen 1995). Under the greater snow accumulation, the shrubs are higher and species such as *Betula nana* L. and *Salix* spp. are intermixed with *V. myrtillus*. Both tundra heath types are characterized by a continuous bryophyte and lichen cover. To date, little is known about how WS and SA habitats differ in microbial community composition and seasonal trends, and the consequences of these changes in community composition on carbon cycling. Soil microbial communities under a thick snow layer with soil temperatures around 0°C have stable conditions that likely enable active soil organic matter (SOM) degradation throughout the winter (Schimel et al. 2004), whereas WS habitats can experience very low temperatures during winter (Niittynen et al. 2020), leading to a need for the community to adjust to such cold conditions. Microbial communities could, thus be expected to be highly divergent between WS and SA tundra habitats.

In this study, we utilized tundra heaths with long-term natural differences in wintertime snow accumulation. The effect of contrasting snow accumulation and subsequent differences in winter soil temperatures on microbial biomass as well as bacterial and fungal community structure were assessed during different seasons. There are indications that the bacterial communities during the growing season are relatively similar in soils of both habitat types and are dominated by stress-tolerant, oligotrophic bacterial taxa such as the Acidobacteriota (Männistö et al. 2013). However, earlier studies of the same tundra sites indicated strong shifts in the relative abundance of dominant bacterial phylotypes in WS heaths especially during spring thaw suggesting that lower winter temperatures and more frequent freeze–thaw cycles may have strong control over the microbial biomass and community structure with a stronger effect in the WS than in deep snow habitats (Männistö et al. 2013). In this study, we evaluated bacterial and fungal biomass using phospholipid fatty acid (PLFA) and quantitative PCR (qPCR) analyses and characterized the community structures by rRNA gene and ITS amplicon sequencing of soil sampled in mid-winter, early, and late growing seasons. The aim was to identify key taxa of the bacterial and fungal communities linked to differences in winter snow accumulation. We hypothesized that (1) differences in the snow accumulation with strong differences in winter soil temperatures lead to divergent patterns in bacterial and fungal biomass between WS and SA tundra heaths during the mid-winter and early growing seasons. We predicted that the more severe freezing and frequent freeze–thaw cycles in WS tundra heaths are associated with lower microbial abundance due to lower microbial activity, and higher turnover, whereas SA tundra heath harbor more stable microbial communities across seasons. We further hypothesized that (2) differences in the winter conditions are associated with differences in the bacterial and fungal community structure, showing a distinct cryotolerant microbial community structure under WS tundra heaths during winter, with only weak differences in the bacterial and fungal communities between the summer and the winter under SA heaths.

## Materials and methods

### Field site

**Figure 1.**
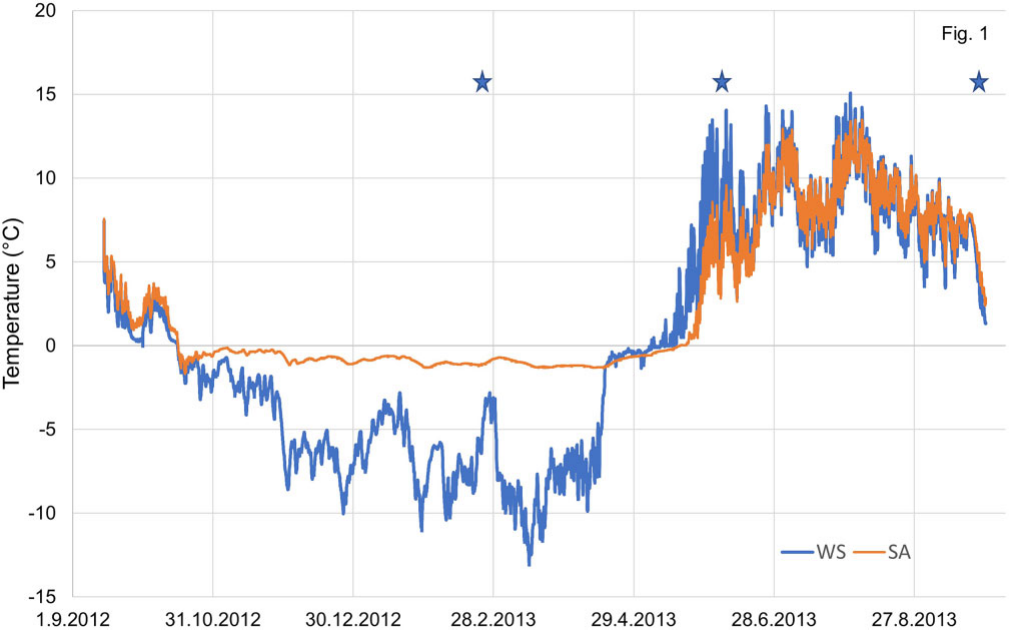
Soil temperature in the WS and SA tundra heaths. Values are means obtained by data loggers in each plot (*N* = 4). Stars denote the sampling points for bacterial and fungal community analyses.

The study site was located in the north side of Mt Pikku-Malla fjeld in Malla Nature reserve, Kilpisjärvi, north-western Finland (69°03′ 50″N, 20°44′40″E). The mean annual precipitation is 420 mm and the mean annual temperature is −1.9°C (Aalto et al. 2018). The bedrock at the sampling sites of this study was formed from siliceous rock materials resulting in acidic barren soil where heaths dominated by the dwarf shrub *E. nigrum* spp. *hermaphroditum* (Hagerup) Böcher prevail (Eskelinen et al. 2009). The tundra heaths are exposed to heavy winds, which together with the differences in topography dramatically influence snow accumulation. Areas with high snow accumulation (up to ≥ 1 m) are located in depressions and areas sheltered from the winds, while WS areas remain essentially snow-free throughout the winter (Figure S1, Supporting Information). Soil temperature under thick snow cover is more stable, remaining close to 0°C throughout the winter, while in the WS heaths, the temperature follows air temperature and may drop down to −15°C (Männistö et al. 2013; Fig 1). In addition to soil temperature and snow accumulation, variations in topography result in alternating patterns in vegetation. *Empetrum nigrum* dominated especially in the WS heaths while *Vaccinium myrtillus* was more abundant in the SA heaths. *Vaccinium vitis-idea* and *Vaccinium uliginosum* were common in both habitat types. Under the higher snow accumulation, the shrub height was higher and species such *B. nana* L. and *Salix* spp. were abundant. Both tundra heath types were characterized by a continuous bryophyte and lichen cover.

### Soil sampling

Four plots (2 m × 2 m) representing WS slopes (i.e. dominated by *E. hermaphroditum*) and four plots corresponding to SA biotopes (i.e. dominated by *V. myrtillus*) were selected and marked based on earlier snow cover estimates (Männistö et al. 2013) and the type of vegetation. All plots were within 300 m from each other and at least 25 m apart. Soil temperature was recorded once every hour using Hobo U10 temperature loggers (Onset Computer, Bourne, Massachusetts) that were buried 3–5 cm below the soil surface. The soil was sampled from WS and SA tundra heaths in February, June, and September 2013 from the top 5 cm (humus layer) using a soil corer (diameter ca. 2 cm). Composite soil samples of five soil cores were taken from each plot. In June and September, the samples were sieved in the field using a 2-mm mesh and immediately frozen in a liquid nitrogen dry shipper. February samples were transported frozen to the lab and sieved after brief thawing. Three subsamples (0.3 g) were taken from each composite sample and stored at −80°C until thawed for DNA/RNA extraction.

### Soil physico-chemical analyses

The dry matter content of the soil was determined by drying the samples (105°C, 12 h) and OM content was analyzed by loss on ignition (475°C, 4 h). Soil pH was measured in 1:5 (v:v) soil:water suspensions (Denver Instrument Model 220). A subsample of ∼3 g fresh soil was extracted for 2 h with 50 ml of 0.5 M K_2_SO_4_. Dissolved organic carbon (DOC) concentrations in these extracts were analyzed with a TOC-VCPH/N Total Organic Carbon Analyzer (Shimadzu Corporation, Kyoto, Japan). Total nitrogen, NH_4_-N and NO_3_-N concentrations were analyzed via flow injection analysis (Quickchem 8000 FIA Analyzer, A83200, Zellweger Analytics, USA). Extractable organic nitrogen (N) was calculated by subtracting inorganic N concentrations from the total. Microbial C and N were extracted from the samples using 0.5 M K_2_SO_4_ after chloroform fumigation for 18 h (Brookes et al. 1985), and then analyzed as total extractable N and DOC. Phosphorus was analyzed colorimetrically (Murphy and Riley 1962).

### Nucleic acid extraction and cDNA synthesis

Total genomic DNA and RNA were extracted from ~0.25 g of soil with slight modifications as described earlier (Männistö et al. 2016) using a CTAB-based method by Griffiths et al. (2000). Three replicate extractions were processed from each of the eight plots. Hexadecyltrimethylammoniumbromide (CTAB; 650 µl) extraction buffer and phenol–chloroform–isoamyl alcohol (25:24:1; pH 8.0; 650 µl) were added together with a mixture of beads to the sample tubes followed by bead beating on a Precellys 24 Dual homogenizer (Bertin Technologies, Montigny-le-Bretonneux, France) for 30 s at 5500 r m^−1^. The bead mixture contained 0.1 mm glass beads (0.3 g), 1.0 mm ceramic beads (0.7 g), and two large (3.5 mm) glass beads (Bio Spec Products Inc., Bartlesville, OK, USA). Samples were further processed as described in Männistö et al. (2016). DNA samples were treated with RNAse A (Thermo Scientific, Waltham, MA, USA) and RNA samples with DNAse I (Thermo Scientific) and converted to cDNA using the Revert Aid H Minus First Strand cDNA Synthesis kit (Thermo Scientific). All solutions used for RNA extraction were treated with 0.1% diethylpyrocarbonate. RNA and DNA concentrations were measured using a Qubit fluorometer and Quant-iT RNA and dsDNA HS assay kits (Thermo Scientific), respectively.

### Microbial PLFA analyses and qPCR

PLFA and qPCR analyses were used to estimate bacterial and fungal biomass in the tundra soil. Microbial lipids were extracted from ca. 1 g (wet weight) of freeze-dried soil as described previously (Männistö et al. 2016). The PLFA 18:2ω6c was used to indicate fungal biomass (including saprotrophic, ectomycorrhizal, and ericoid mycorrhizal fungi; Olsson 1999, Ruess et al. 2002), while the sum of PLFAs i15:0, a15:0, 15:0, i16:0, 16:1ω9c, i17:0, a17:0, 17:0, cyclo-17:0, 18:1ω7c, and cyclo-19:0 was used to indicate bacterial biomass (Frostegård and Bååth 1996).

qPCR was performed using the Bio-Rad CFX96 Real-time thermal cycler (Bio-Rad) and SsoAdvanced Universal SYBR Green Supermix (Bio-Rad). 16S rRNA gene copy numbers were quantified using the primer pair Eub341F (CCTACGGGAGGCAGCAG) and Eub534R (ATTACCGCGGCTGCTGG) (Muyzer et al. 1993), and fungal ITS2 region copies with the primer pair fITS7 (GTGARTCATCGAATCTTTG) (Ihrmark et al. 2012) and ITS4 (TCCTCCGCTTATTGATATGC) (White et al. 1990). All qPCRs were run in technical triplicates of 20 µl and contained 10 µl Supermix, 0.5 µl of each primer (10 mM), 7 µl ddH2O, and 2 µl template in a 100-fold dilution. PCR conditions for bacterial analysis were 98°C for 2 min followed by 40 cycles of 98°C (5 s), 56°C (20 s), and for fungal analysis 98°C for 3 min followed by 40 cycles of 98°C (15 s), 61°C (60 s), (following a plate read). Genomic DNA from *Granulicella mallensis* MP5ACTX8 isolate was used as a bacterial and *Laccaria laccata* isolate as a fungal standard.

### PCR and Ion Torrent sequencing

First amplification of the V1–V3 region of the 16S rRNA gene was done in duplicates for each DNA/cDNA sample in 20 µl reactions containing 1 µl of 1:50 diluted DNA template, DreamTaq DNA Polymerase (Thermo Scientific), 0.3 µM of each primer (27F 5′-AGAGAGTTTGATCMTGGCTCAG-3′; Lane 1991, and 518R 5′-ATTACCGCGGCTGCTGG-3′, Muyzer et al. 1993), 3.2 µg of bovine serum albumin and 0.2 mM of dNTP mix. In the second amplification step, primer IonA_bc_27F included adapter IonA (5′-CCATCTCATCCCTGCGTGTCTCCGAC-3′) and unique 10–12 bp long barcode sequences before the primer 27F and primer P1_518r included P1 5′-CCTCTCTATGGGCAGTCGGTGAT-3′ before the primer 518r to allow Ion Torrent sequencing and assignment to specific samples. The cycling regime for the first PCR included denaturation of 95°C for 5 min, followed by 25 cycles of 94°C 30 s, 55°C 30 s, and 72°C 1 min, and a final elongation step of 72°C for 10 min. The second PCR reaction contained 1 µl of the reaction product of first amplification, and only 15 cycles were run. PCR products were cleaned using the Agencourt AMPure XP magnetic beads purification system (Beckman Coulter, Brea, CA, USA) and quantified with Qubit dsDNA HS Assay Kit (Thermo Scientific). Amplicons were then combined in equimolar concentrations for sequencing. The pooled 16S rRNA gene amplicon libraries were sequenced using Ion Torrent Personal Genome Machine (Thermo Scientific) at the University of Jyväskylä, Finland. One set of samples (DNA samples of February) was sequenced in 2014 using a 316 v2 chip and all other samples in 2015 using a 314 v2 chip. The sequencing chemistry and Ion Torrent software was updated multiple times between these two runs, potentially affecting the results, and therefore the bacterial DNA sequences from February were not compared with the other sampling seasons (see the section “Statistical Analyses”). Amplification of the ITS2 region of fungal rRNA operons was performed as a 2-step procedure recommended by Berry et al. (2011). The first amplification step was done in triplicate for each sample (1 µl of 1:50 dilution) in a 10-µl reaction and the second step in a single 50 µl reaction for each sample (1 µl of PCR product from step 1), both amplification steps using Phusion High-Fidelity DNA Polymerase (Thermo Scientific), Phusion HF Buffer, and 0.2 mM of dNTPs and each primer (ITS7 5′-GTGARTCATCGAATCTTTG-3′, Ihrmark et al. 2012 and ITS4 5′-TCCTCCGCTTATTGATATGC-3′, White et al. 1990). In the second amplification step, ITS7 primer included the adapter P1 5′-CCTCTCTATGGGCAGTCGGTGAT-3′ and ITS4 primer included the adapter IonA 5′-CCATCTCATCCCTGCGTGTCTCCGACTCAG-3′ and unique 10-bp long barcode sequences at the beginning of the primer to allow Ion Torrent sequencing and assignment to specific samples. The cycling regime for the first step was: initial denaturation of 98°C for 1 min, followed by 25 cycles of 98°C 10 s, 54°C 20 s, and 72°C 30 s, and a final elongation step of 72°C for 7 min. For the second step cycling regime was the same as in the first except for changing the cycle number to 10 and the annealing temperature to 57°C. Fungal ITS amplicons were sequenced at Biocenter Oulu Sequencing Center (Univ. Oulu, Oulu, Finland). PCR products were first cleaned using the Agencourt AMPure XP magnetic beads purification system and BioMek4000 Laboratory Automation Workstation (Beckman Coulter) followed by purity checking and quantification using MultiNA (MultiNA Microchip Electrophoresis System and DNA-1000 kit; Shimadzu, Kyoto, Japan) and PicoGreen (Thermo Scientific) according to manufacturers’ instructions. Amplicons were then combined in equimolar concentrations for Ion Torrent PGM sequencing with 314 v2 chip.

### Bioinformatics

Bacterial sequence reads from the two sequencing efforts (total 3 005 260 and 2 666 670 reads) were demultiplexed, quality filtered and merged with the following adjustments using QIIME 1.9.1 (Caporaso et al. 2010): minimum length of 300 bp, maximum of one mismatch in primer sequences, and minimum mean quality score of 25 within 50 bp window size. We employed a reference-based operational taxonomic unit (OTU) picking against SILVA release 128 99% identity reference database (Quast et al. 2013), removing chimeras, and clustering all sequences using USEARCH 6.1 (Edgar 2010, Edgar et al. 2011) with a sequence similarity value of 97%. Taxonomy was assigned for representative sequences using a naïve Bayesian RDP classifier (Wang et al. 2007) against SILVA 99% identity majority taxonomy strings with a confidence threshold of 50%. Representative sequences from each OTU were aligned to the SILVA 99% identity reference alignment using PyNAST (Caporaso et al. 2009) and a phylogenetic tree was built using FastTree (Price et al. 2009). After removing singletons (OTUs represented by a single sequence) and alignment failures from the data, 1 249 249 reads were obtained from all samples with an average of 10 498 reads per sample (min 3314 and max 32 820). For downstream analyses, all samples were rarefied by random sampling (without replacement) to an equal sequence number of 3300 to minimize bias due to different sequencing efforts across samples.

ITS sequence reads (total 701 430 reads) were demultiplexed and quality filtered with the following adjustments using QIIME 1.9.1 (Caporaso et al. 2010): minimum length of 200 bp, the maximum length of 600 bp, maximum of one mismatch in primer sequences, and minimum mean quality score of 20 within 50 bp window size. We employed a chimera check using UCHIME (Edgar et al. 2011) and sequences were then clustered into OTUs using UCLUST (Edgar 2010) with a sequence similarity value of 97%. Taxonomy was assigned for representative sequences using BLAST (Altschul et al. 1990) against UNITE v.7.1 97% threshold reference database (Kõljalg et al. 2013) with 90% identity. After removing singletons (OTUs represented by a single sequence) and nonfungal hits from the data, 404 561 reads were obtained from all samples with an average of 6321 reads per sample (min 1486 and max 10 579). For downstream analyses, all samples were rarefied by random sampling (without replacement) to an equal sequence number of 1400 to minimize bias due to different sequencing effort across samples.

Raw sequence data and associated metadata were deposited in GenBank with the Bioproject accession no. PRJNA1080106.

### Statistical analyses

Differences in bacterial and fungal community structure were analyzed using permutational analysis of variance (PERMANOVA; Anderson 2001) and visualized with principal coordinate ordination (PCO). For the PERMANOVA and PCO analyses, bacterial and fungal OTU data were Hellinger-transformed and Bray–Curtis dissimilarity matrices used as the resemblance matrices. Habitat (WS, WS, or SA) and sampling season (winter, early, or late growing season) were used as fixed factors and plot as a random factor nested in habitat. When significant interactions were detected for habitat and season, pairwise PERMANOVA was performed on the respective terms. All PERMANOVA analyses were performed with 999 random permutations PERMANOVA and ordination analyses were performed using the PERMANOVA+ add-on (Anderson et al. 2008) for PRIMER v7 (Clarke and Gorley 2015). Data analysis indicated that bacterial communities of winter DNA samples deviated strongly from all other samples (derived from DNA or RNA) as illustrated by a PCO ordination (Figure S2, Supporting Information). This deviation may be, at least partially, due to a sequencing bias as the winter DNA-derived bacterial communities were sequenced earlier than the other samples. Samples of the different sequencing runs were sequenced using a different chip and sequencing chemistry and the also the server software was updated between the runs. Therefore, to avoid potential sequencing bias the February DNA data were not compared with other sampling points or with RNA-derived community structure in the statistical analysis.

The effect of habitat, sampling season, and their interaction on microbial biomass proxies (16S rRNA gene and ITS copies, total PLFAs, bacterial PLFAs, fungal PLFA, and bacterial/fungal PLFA ratio) and soil physico-chemical properties as well as on the abundance of 10 most abundant bacterial and fungal genera were tested using linear mixed effect model (LME) with habitat and season as fixed factor and plot as random factor. Sampling season was assigned as repeated factor with site as a subject and AR1 as the covariance structure. When significant interactions were detected for habitat and season, habitats were further tested separately with a least significant difference test as a *post hoc* test under the linear mixed model. Logarithmic transformations were used as necessary to meet the assumptions the linear mixed model. LME tests for microbial biomass and soil parameters were conducted using IBM SPSS 29.0 software. Distance based linear modelling (DistLM) (Legendre and Anderson 1999, Anderson et al. 2008) was used to determine the extent to which soil variables explain bacterial and fungal community structure in WS and SA tundra heaths. Multicollinearity between variables was first tested using the Draftsman Plot function and spearman correlations in PRIMER v7 (Clarke and Gorley 2015) and from variables that correlated by more than 0.9, only one was picked. Of the nitrogen forms, total and organic N correlated by 0.96 and N_org_ was, therefore omitted from the analysis. Logaritmic transformations were used for the same variables as for the LME tests (N_tot_, NH_4_, P_tot_, and P_mic_). Marginal tests identified the influence of individual soil variables on bacterial (DNA and RNA) and fungal community structure without considering the effect of other variables. To identify the soil parameters that in combination explained bacterial and fungal community structure, DistLM model was utilized with stepwise selection procedure and corrected Akaike information criterion as the selection criteria with 999 permutations. The resulting models were visualized using distance-based redundancy analysis (dbRDA) plots. DistLM analyses and dbRDA plots were done performed using the PERMANOVA+ add-on (Anderson et al. 2008) for PRIMER v7 (Clarke and Gorley 2015).

## Results

### Soil physico-chemical properties and microbial biomass in WS and SA tundra heaths

Soil temperature differed strongly in WS vs. SA tundra (Fig. 1). The average soil temperature from September 2012 to September 2013 was 0.1°C in the WS plots and 2.4°C in SA plots. During the coldest months (December–March), the average soil temperature was −7.1°C and −1.0°C in the WS and SA plots, respectively. On the other hand, due to earlier spring thaw and less insulating vegetation cover, the WS plots were warmer in June with an average soil temperature of 8.4°C compared to 7.0°C in the SA plots.

LME model indicated that there were no significant differences in soil pH, moisture, or OM content between WS and SA heaths, but they differed in nutrient availability. Total nitrogen (N_tot_, *F* = 21.34, *P* = .004), organic nitrogen (N_org_, *F* = 17.053, *P* = .007), NH_4_-N (*F* = 7.534, *P* = .034), and total phosphorus (P_tot_, *F* = 10.675, *P* = .017) availability were significantly higher in the SA than WS heaths throughout the sampling year. On the other hand, N stored in microbial biomass (N_mic_, *F* = 5.353, *P* = .060) tended to be higher in the WS than under SA heaths. Sampling season had a significant impact on soil N availability, the concentrations of all N forms were at their lowest in samples collected in late growing season (Table 1; Table S1, Supporting Information). However, when the habitats were tested separately, sampling season had a significant effect on N_tot_ only in the WS heaths (*P* = .012 between winter and early growing season and *P* < .001 between other sampling seasons), while NO_3_ availability was significantly different between winter and early growing season (*P* = .008) and early and late growing season (*P* = .001) in WS heaths, and winter and early growing season (*P* = .049) and winter and late growing season (*P* = .003) in SA heaths.

**Table 1.** Soil physico-chemical properties in WS and SA tundra heaths. Values are means with SE in brackets.

	Winter		Early GS		Late GS	
	WS	SA	WS	SA	WS	SA
pH	4.6 (0.04)	4.5 (0.03)	4.7 (0.04)	4.6 (0.04)	4.6 (0.03)	4.4 (0.07)
OM (%)	75.4 (4.7)	68.1 (6.5)	74.2 (5.0)	69.4 (3.1)	73.6 (5.5)	69.0 (4.2)
DW (%)	35.1 (2.5)	37.2 (2.6)	36.1 (2.4)	32.0 (1.2)	32.6 (2.4)	32.4 (1.7)
N_tot_ (mg kg^−1^)	45.9 (3.1)	94.4 (10.0)	53.8 (2.4)	100.4 (15.3)	32.9 (1.5)	81.3 (6.7)
N_org_ (mg kg^−1^)	43.9 (3.0)	78.1 (7.8)	50.1 (2.3)	90.9 (13.0)	30.8 (1.4)	74.8 (5.6)
NO_3_ (mg kg^−1^)	0.25 (0.02)	0.47 (0.08)	0.16 (0.01)	0.26 (0.02)	0.27 (0.02)	0.19 (0.05)
NH_4_ (mg kg^−1^)	1.74 (0.13)	15.82 (6.0)	3.53 (0.26)	9.18 (2.3)	1.82 (0.26)	6.33 (1.2)
N_mic_ (mg kg^−1^)	158.4 (11.7)	129.3 (8.4)	130.4 (12.5)	93.8 (6.4)	125.0 (5.1)	92.1 (6.0)
P_tot_ (mg kg^−1^)	15.1 (1.9)	52.8 (14.3)	13.3 (1.8)	43.2 (4.6)	10.0 (0.83)	32.3 (8.7)
P_mic_ (mg kg^−1^)	76.4 (9.9)	58.8 (11.7)	48.5 (11.7)	51.4 (5.7)	45.5 (5.6)	41.5 (8.2)

**Figure 2.**
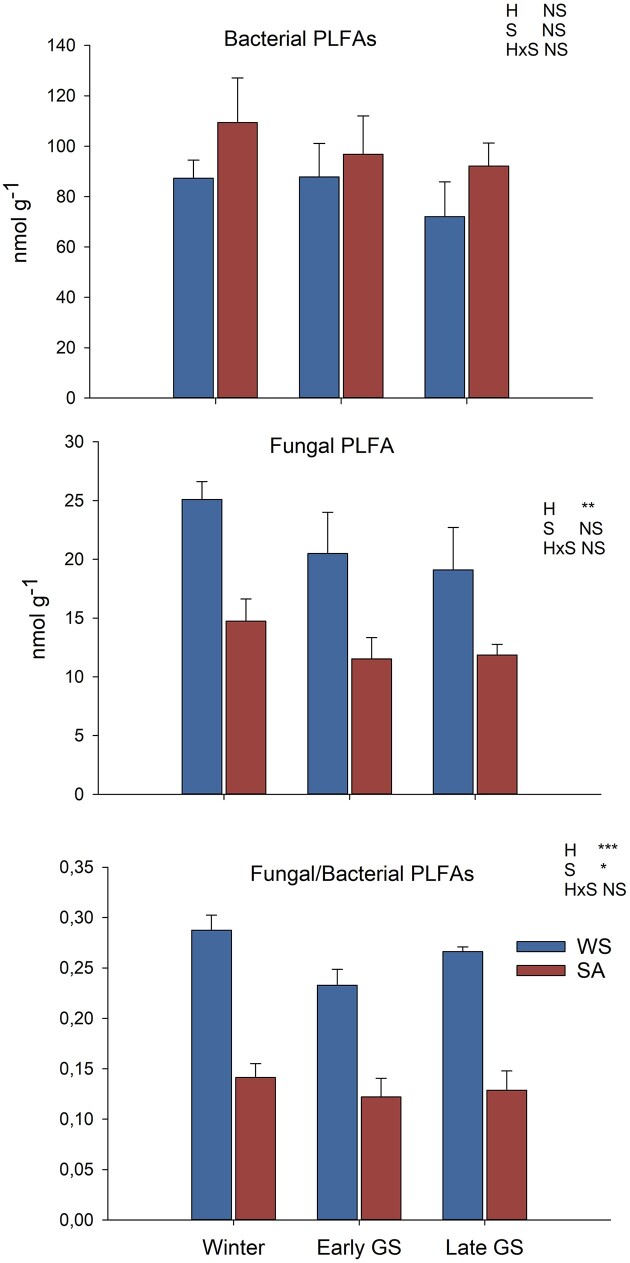
Bacterial and fungal PLFAs and their ratio in WS and SA tundra heaths sampled in winter, early, and late growing season. Values are means ± SE, *N* = 4. Significant differences of between the habitats (H) or sampling season (S) were analyzed using LME model (Table S1, Supporting Information). Significance levels: ***, *P* < .001; **, *P* < .01; *, *P* < .05; and NS, not significant.

**Figure 3.**
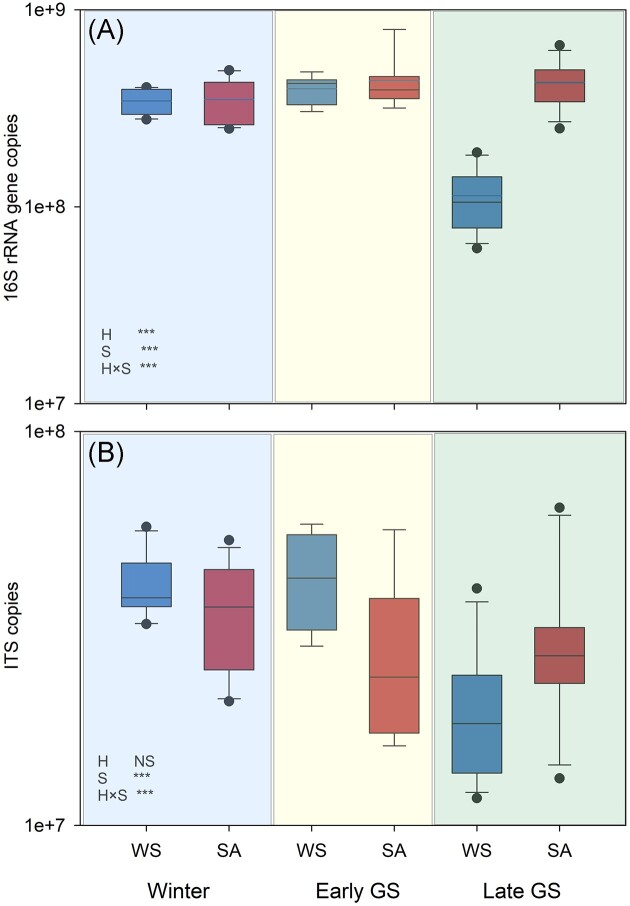
Bacterial 16S rRNA and fungal ITS copy numbers in WS and SA tundra heaths sampled in winter, early, and late growing season. Values are means ± SE, *N* = 4. Significant differences of between the habitats (H) or sampling season (S) were analyzed using LME model (Table S1, Supporting Information). Significance levels: ***, *P* < .001; **, *P* < .01; *, *P* < .05; and NS, not significant.

Microbial biomass was estimated using soil PLFA analysis and qPCR of bacterial 16S rRNA gene and fungal ITS region copy numbers (Figs 2 and 3). The LME model indicated that there were no significant differences in the abundance of total PLFAs between the different tundra heaths or sampling dates. Bacterial PLFAs were higher in the SA than WS plots and these decreased toward the late growing season, but the differences were not statistically significant. The fungal PLFA marker was in higher abundance in the WS heaths (*F* = 16.208, *P* = .007) and tended to be lower (*F* = 2.732, *P* = .086) in the early and late growing season samples. The fungal to bacterial PLFA ratio was higher in WS heaths (*F* = 82.804, *P* < .001) and was lowest during June (*F* = 4.728, *P* = .019) (Fig. 2; Table S1, Supporting Information).

Bacterial 16S rRNA gene copy numbers were significantly different (*F* = 31.165, *P* = .001;) between WS and SA heaths as well as at different sampling seasons (*F* = 38.792, *P* < .001) and there was a significant habitat × season interaction (*F* = 54.388, *P* < .001; Table S1, Supporting Information).This was attributed to the significantly lower copy numbers in the WS plots during late growing season (*P* < .001). LME test indicated no significant main effect of habitat on fungal copy numbers, but when the habitats were tested separately, pairwise tests indicated that in WS heaths, ITS copy numbers were significantly lower *P* < .001) in late growing season than in winter or early growing season (Fig. 3; Table S1, Supporting Information).

### Bacterial community structure in WS and SA tundra heaths

Bacterial community composition was characterized by 16S rRNA gene (representing total community) and reverse transcribed 16S rRNA (representing active community) amplicons. Due to a potential bias associated with the differences in sequencing (see the section “Materials and methods”), winter bacterial DNA samples were excluded from the multivariate statistics (PCO and PERMANOVA) and not compared to data of the other seasons.

**Figure 4.**
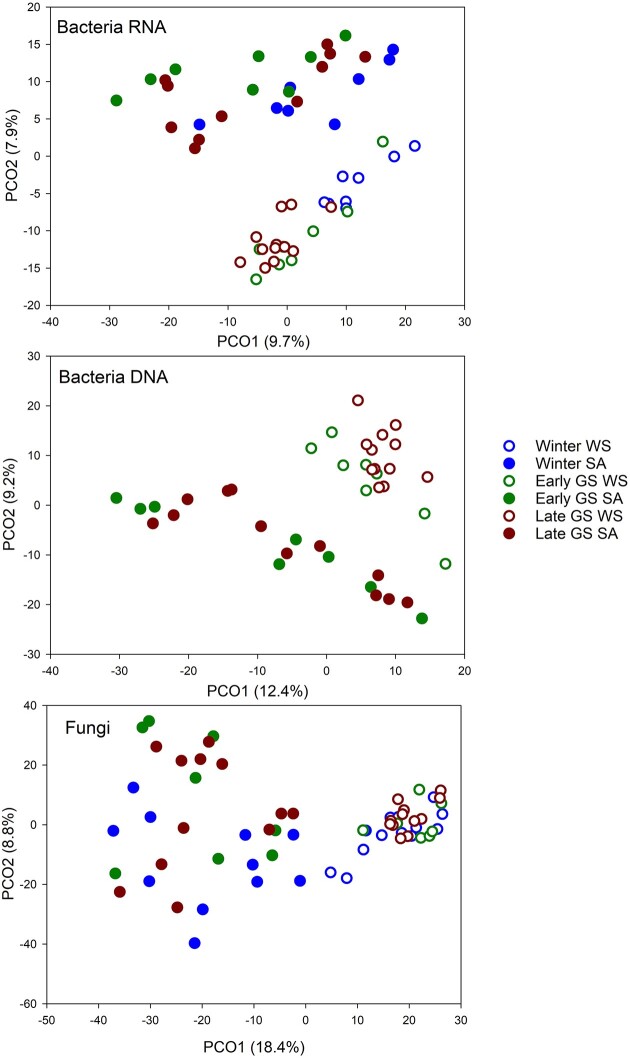
PCO ordination of active (RNA derived) and total (DNA derived) bacterial communities and total fungal communities in WS and SA tundra heaths. Winter DNA-derived bacterial communities are excluded from the ordination due to possible technological bias in the data (see the section “Materials and methods”).

PCO ordination indicated the greatest differences between the DNA (total) and RNA (active) derived bacterial community structures (Figure S3, Supporting Information), which separated the samples into two main groups along the first axis that explained most of the variance. To delineate the effects of habitat and sampling season on the total vs. active communities, the DNA and RNA-derived datasets were analyzed separately. PCO ordination grouped both total and active communities from WS and SA tundra heaths separately (Fig. 4). This was supported by PERMANOVA analysis in which habitat explained slightly more of the community variation than sampling season (Table 2). Pairwise PERMANOVA analysis for the WS and SA habitats separately indicated that the active bacterial communities were significantly different in all sampling seasons (*P* = .001–.004) except in SA heaths between early and late growing season. Total bacterial communities were different in WS heaths (*P* = .001) but not in SA heaths (*P* = .265) between early and late growing season.

**Table 2.** PERMANOVA main test for bacterial (derived from RNA and DNA) and fungal community structure in WS and SA habitats (Ha) across three different seasons (Se) in four plots per habitat. Significance levels: *P* < .05 are underlined, *P* < .01 are indicated by bold.

Source of variation	SS	MS	Pseudo- F	*P* (perm)	Variance explained (%)
**Bacteria RNA**					
Habitat	4726	4726.4	2.33	**.01**	14.6
Season	5032	2516.1	2.40	**.001**	12.8
Plot (Ha)	14 865	2123.5	2.02	**.001**	18.5
Se × Ha	2955	1477.6	1.41	**.001**	9.8
**Bacteria DNA** **^a^**					
Habitat	4903	4902.7	2.43	.018	17.5
Season	1613	1613.1	1.57	**.001**	8.2
Plot (Ha)	14 460	2065.7	2.02	**.001**	21.6
Se × Ha	1331	1331.4	1.30	.047	8.4
**Fungi DNA**					
Habitat	19 501	19 501	4.15	**.002**	21.5
Season	7428	3713.7	2.30	**.001**	9.7
Plot (Ha)	34 990	4998.6	3.10	**.001**	20.7
Se × Ha	5969	2984.4	1.85	**.001**	11.1

a For the bacterial DNA PERMANOVA analyses, only samples from early and late growing season were included.

**Figure 5.**
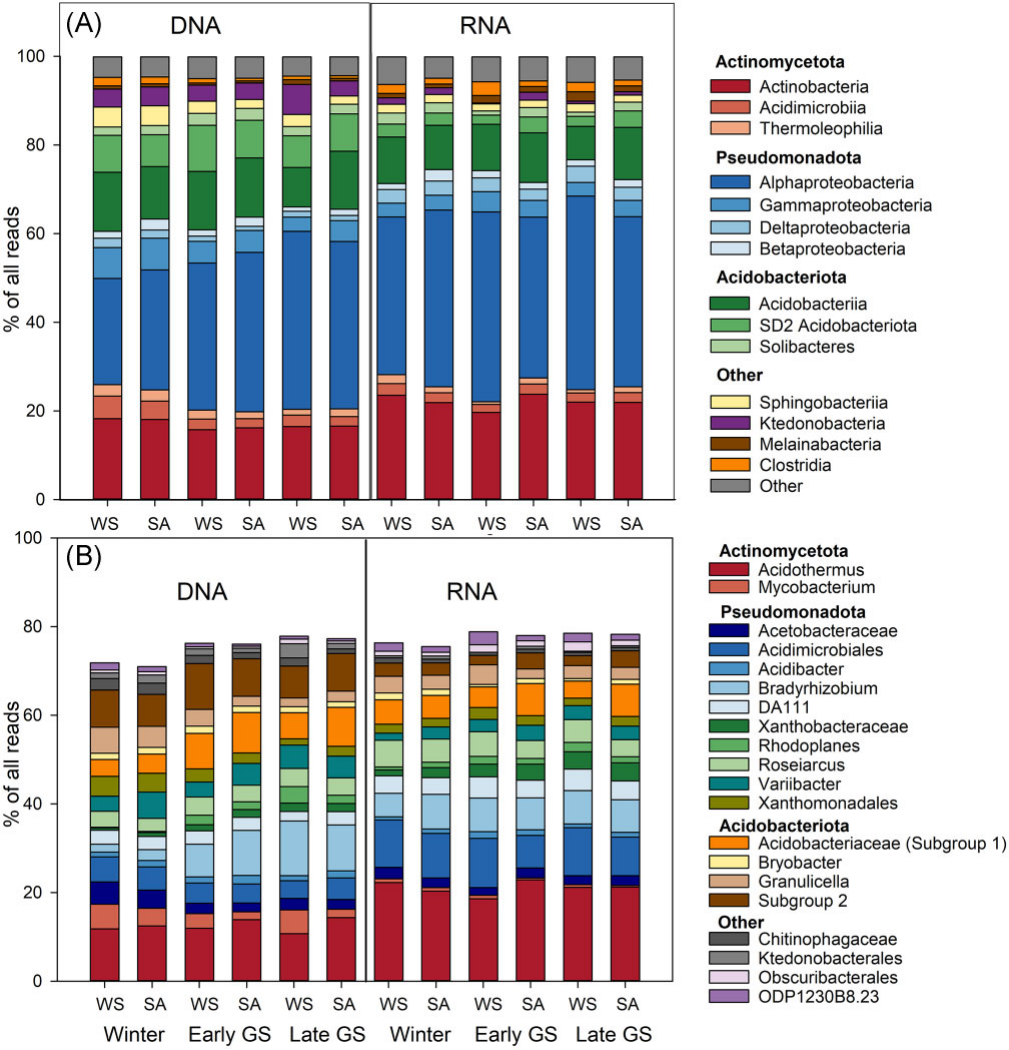
Abundance of bacterial classes (A) and dominant genera (B) in WS and SA tundra heaths sampled in winter, early, and late growing season.

Both tundra heath types were dominated by Actinomycetota, Pseudomonadota (mainly class Alphaproteobacteria), and Acidobacteriota (Fig. 5). At the genus level, the most dominant Actinomycetota were *Acidothermus* spp., *Mycobacterium* spp., and unknown taxa of the class Acidimicrobiales. The most abundant Acidobacteriota were *Granulicella* spp., *Bryobacte*r spp., *Edaphobacter* spp., unknown Acidobacteriia (subdivision (SD) 1), and unknown SD 2 Acidobacteriota. The most abundant Pseudomonadota were unknown members of the Acetobacteraceae, *Roseiarcus* spp., *Bradyrhizobium* spp., *Variibacter* spp., *Rhodoplanes* spp., unknown genera of Caulobacteraceae and Xanthobacteraceae, and the DA111 group of Alphaproteobacteria. The same genera dominated both in the DNA and RNA amplicons but with differences in the relative abundances (Fig. 5; Figure S3, Supporting Information). Of the Alphaproteobacteria, unknown genera within the Acetobacteraceae and Xanthobacteraceae were more abundant in the RNA-derived community while *Bradyrhizobium* spp. were more abundant in the DNA. Similarly, *Acidothermus* spp. (Actinomycetota) were significantly more abundant in RNA while *Mycobacterium* spp. were more abundant in the DNA-derived community. Of the Acidobacteriota, unknown members of SD2 and SD1 were more abundant in the DNA- than RNA-derived community.

Comparison of the active bacterial communities between WS and SA tundra heaths revealed relatively small differences in the dominant bacterial groups (Fig. 5; Figure S4, Supporting Information). Of the 10 most dominating active bacterial genera, unknown genera within the family Acetobacteraceae and members of Acidobacteriota were significantly affected by the habitat (LME, *P* < .05), but this interacted with the season. Acetobacteraceae were more abundant in WS heaths only in early and late growing season. Of the Acidobacteriota, unknown Acidobacteriaceae were more abundant in the SA tundra heaths in the early and late growing season, *Granulicella* spp. were more abundant in WS tundra heaths but only in the early growing season, while members of SD2 Acidobacteriota were more abundant in SA tundra in the late growing season. The abundance of different genera of Pseudomonadota was generally more affected by sampling season than the habitat (Figure S4, Supporting Information).

### Fungal communities in WS and SA tundra heaths

**Figure 6.**
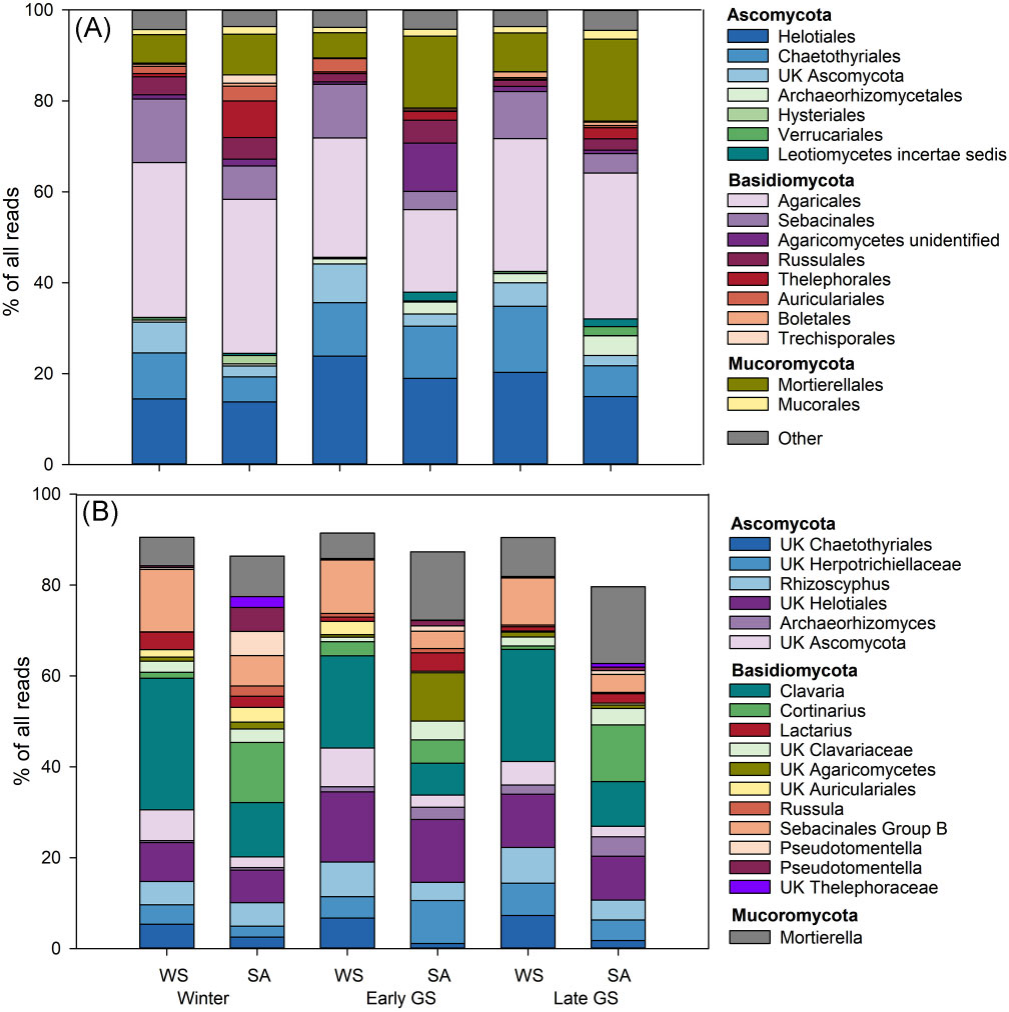
Abundance of fungal orders (A) and dominant genera (B) in WS and SA tundra heaths sampled in winter, early, and late growing season. UK = unknown.

PCO and PERMANOVA analyses indicated that fungal communities were structured more by the habitat than by sampling season (Fig. 4 and Table 2). Moreover, the communities were more dispersed in the SA than WS heaths. Pairwise PERMANOVA analysis indicated that there were no differences between winter and early growing season community structure in the WS tundra heaths while all other sampling seasons differed in WS and SA heaths (*P* = .001–.049). Fungal communities under both tundra heaths were dominated by Basidiomycota, Ascomycota, and Mucoromycota. Basidiomycota were the most abundant fungi under both WS and SA heaths and were especially dominant in the winter samples. Of the Basidiomycota, the order Agaricales dominated in both habitats with *Clavaria* spp. as the most dominant genus in WS heaths and *Cortinarius* spp. more abundant in the SA heaths. Group B Sebacinales were more associated with the WS heaths. Members of the order Helotiales were the dominant Ascomycota both in WS and SA heaths while members of Chaetothyriales were more abundant in the WS heaths (Fig. 6).

Of the 10 most abundant genera, *Clavaria* spp., unknown Sebacinales, unknown Chaetothyriales, and unknown Ascomycota were significantly (LME; *P* < .05) more abundant in the WS tundra heaths while *Mortierella* spp. were more abundant in the SA heaths. Members of *Cortinarius* were more abundant in the SA heaths, but due to the high variation between samples, the difference was not significant. *Rhizoscyphus* and other Helotiales were abundant in both heath types and tended to be more abundant in the WS heath, but the difference was not statistically significant (Figure S5, Supporting Information).

### Effect of soil physico-chemical properties on bacterial and fungal community structure

Distance based linear modeling was used to determine the contribution of soil factors on bacterial and fungal community structures. Marginal tests indicated that except for NO_3_, all nine tested soil factors had a significant impact on both bacterial and fungal community structure (Table S2 and Figure S6, Supporting Information). Total nitrogen explained most of the variation of bacterial and fungal community structure and was also among the variables in the best DistLM model that predicted the community structure. Active, RNA-derived bacterial community structure was predicted best by N_tot_, P_mic_, OM%, and N_mic_. while total, DNA derived bacterial community (with only early and late growing season samples) was predicted by pH, N_tot_, and N_mic_ and fungal community by Ntot, Nmic, pH, and OM% (Figure S6, Supporting Information). The models explained 20.6%, 20.0%, and 26.3% of the variation in RNA-derived, DNA-derived bacterial and fungal community structure, respectively.

## Discussion

WS and SA tundra heaths are characterized by large differences in soil temperature. In addition to strong differences in the winter soil temperatures, there is a difference in the amplitude of annual temperature variation. Due to earlier snowmelt and lower insulation from vegetation, the WS heaths are associated with high-temperature fluctuation and, depending on the air temperatures, also prone to frequent freeze–thaw cycles during spring (Männistö et al. 2013; this study). These differences in topography lead to a spatially heterogeneous snow cover depth and soil temperatures that in turn form predictable patterns in the dominant vegetation (Oksanen and Virtanen 1995, Niittynen et al. 2020), but so far, how these differences drive microbial community composition has remained uninvestigated. Supporting our hypothesis that the topographic differences and associated consequences on vegetation, soil temperatures, nutrient availability as well as the quantity and quality of OM modify soil microbial community composition, PLFA, qPCR, and sequencing of 16S rRNA genes and the ITS region indicated distinct differences in the bacterial and fungal communities in WS vs. SA tundra heaths. Contrasting our hypothesis, however, the seasonal trends were either uniform between the WS and SA tundra heaths or amplified toward the end of the growing season rather than being greatest in winter when the difference in soil temperatures was the greatest. These results imply that differences in the bacterial and fungal communities in WS and SA tundra heaths across different seasons were likely driven by vegetation, soil nutrients, and carbon substrates rather than by soil temperature *per se*.

PLFA and qPCR analyses indicated generally higher fungal abundance in the WS tundra heaths while bacterial PLFAs tended to be higher in the SA tundra heaths with higher nutrient availability. This is in line with other studies from tundra showing positive correlations of bacterial abundance with nutrient availability (Eskelinen et al. 2009, Stark et al. 2012). Contrary to our hypothesis, there were only minor differences in the bacterial and fungal gene copy numbers between WS and SA heaths in winter, but differences were magnified toward the end of the growing season when both bacterial and fungal gene copy numbers dropped in the WS heaths, coinciding with drastically lower soil N and P concentrations. The parallel trends in bacterial and fungal gene copy numbers and nutrient availability indicate that limitations in soil nutrient availability may have contributed to increased microbial turnover during the growing season in WS heaths, which supports earlier findings of strong competition for nutrients between plants and microbes in Arctic nutrient-poor soils (Jonasson et al. 1996, Stark and Kytöviita 2006, Stark et al. 2023). Owing to a strong nutrient limitation, tundra soil carbon and nitrogen cycles are strongly coupled, and microbial N immobilization and even soil microbial biomass may be regulated by plant nitrogen uptake (Jonasson et al. 1999, 2001, Schimel and Bennett 2004). Following the patterns of plant nitrogen uptake, soil nitrogen availability often decreases during the growing season, leading the soil microbial communities to become increasingly N-limited toward the end of the growing season (Weintraub and Schimel 2005a,b, Stark and Kytöviita 2006, Wallenstein et al. 2009). This induces a strong seasonality of microbial biomass and activities that may be at their highest in early spring and late autumn when the plant activity is at its lowest (Stark and Kytöviita 2006, Stark and Väisänen 2014). The decrease of bacterial and fungal gene copy numbers toward the end of the growing season in the nutrient-poor WS tundra heaths is, thus in line with microbial communities being driven more by nutrient limitations than directly by the winter temperatures.

Another factor that may have contributed to the decline in microbial biomass in WS tundra heaths toward the end of the growing season could be decreased availability of labile C that would increase turnover of microbial biomass. In contrast, some studies suggest that increased wintertime decomposition under increased snow depth may decrease microbial respiration in the following growing season due to reduced availability of labile C substrates (Semenchuk et al. 2016, Sullivan et al. 2020). Differences in vegetation between WS and SA sites, however, likely cause differences in soil C and N by multiple mechanisms. *Empetrum nigrum* that was especially dominant in the WS sites produce allelophatic compounds and slowly decomposable litter that generally decelerate soil nutrient and carbon cycles (Bråthen et al. 2010, Vowles and Björk 2019). Moreover, the known and putative ErM fungi, which were more dominant in the WS sites, produce recalcitrant necromass that further increases the stability of the OM and reduces the availability of labile C and N forms (Clemmensen et al. 2015, 2021). Snow-cover related to topography may, thus be an important microclimatic driver of both microbial community composition and SOM dynamics.

The higher fungal PLFA abundance and fungal-to-bacterial ratio in the WS tundra heaths (Table S1, Supporting Information) was associated with clear differences between the fungal communities in WS and SA tundra heaths. While known ericoid mycorrhizal fungi, such as *Rhizoscyphus* spp. and other *Helotiales* (Clemmensen et al. 2015, Leopold et al. 2016) were abundant in both, WS and SA tundra heaths (Fig. 6), ectomycorrhizal fungi, especially the genus *Cortinarius*, were more abundant in some sites of the SA tundra heaths corresponding to higher abundance of ectomycorrhizal dwarf shrubs (*B. nana* and *Salix* spp.) under SA heaths. The low abundance of *Cortinarius* spp. in WS heaths may, however, also be explained by the sensitivity of these fungi to freezing (Ma et al. 2011) as some *Cortinarius* species have been shown to benefit from increased snow depth in dry tundra sites of Alaska (Morgado et al. 2016). Members of the genus *Clavaria* were the most abundant fungal taxa at the genus level and significantly more abundant in the WS tundra heaths. The most abundant OTU of the whole fungal data set (> 11% of all sequence reads) was related to *Clavaria* sequences from Arctic soils (Deslippe et al. 2012, Dahl et al. 2017) and *Clavaria argillacea* sampled from Greenland, indicating an association to Arctic ecosystems. *Clavaria* has been reported as the dominant taxa in tundra soils of Alaska (Semenova et al. 2016), Greenland (Voříšková et al. 2019), and in Raisduoddar fell in northern Norway located close to our study site (Ahonen et al. 2021). *Clavaria* spp. are considered saprotrophic, but they have been found abundantly in hair roots of ericoid shrubs such as *V. uliginosum* (Yang et al. 2018), *Vaccinium corymbosum* (Li et al. 2020) and bidirectional nutrient transport between a *Clavaria* and ericaceous plant species have been reported (Englander and Hull. 1980), indicating that they may have symbiotic associations with the ericoid shrub vegetation in nutrient-poor tundra heaths. *Clavaria* was identified as one of the most dominant taxa associated with roots of the Ericaceae shrub *Cassiope tetragona* growing in Svalbard (Lorberau et al. 2017), further suggesting its importance in Ericaceae shrub-dominated tundra heaths. The higher abundance in the more nutrient-poor WS tundra heaths in this study suggests a role of *Clavaria* spp. in nutrient accessibility and transport between/for the ericoid vegetation. Furthermore, members of *Clavaria* and Clavariaceae have been associated with freeze–thaw tolerant fungal communities in soil from Northern Sweden (Perez-Mon et al. 2020), suggesting their resistance to more extreme winter conditions, which may contribute to their abundance in the WS heaths.

Other fungal taxa that were consistently more abundant in the WS tundra heaths were members of the order Chaetothyriales and the family Serendipitaceae of the order Sebacinales (group B). Sebacinales comprises a diverse group of cryptic organisms that are considered to form symbiotic relationships with many types of vegetation (Leopold et al. 2016, Weiß et al. 2016), including ericoid mycorrhizal associations with Ericaceae shrubs (Selosse et al. 2007, Vohník et al. 2016). Similar to *Clavaria*, Sebacinales was reported as the dominant order of the roots of *C. tetragona* and *Bistorta vivipara* in Svalbard where they were suggested to play an important role in the Arctic tundra ecotone either as mycorrhizae or as endophytes (Blaalid et al. 2014, Lorberau et al. 2017). However, the ecological role of the Sebacinales in these ecosystems require further research as OTUs both in this study and those from Svalbard had low sequence similarities to known Sebacinales, a taxonomically and functionally a diverse group with many unknown representatives (Oberwinkler et al. 2013).

PERMANOVA analysis indicated significant differences in the bacterial communities between WS and SA tundra heaths during all sampling seasons but contrary to our hypothesis there were relatively minor differences in the dominant bacterial taxa during winter. Bacterial communities were dominated by members of Actinomycetota, Pseudomonadota (Alphaproteobacteria), and Acidobacteriota. These groups have been shown to dominate the soil and mycosphere of ericoid shrubs (Timonen et al. 2017), indicating their link to the shrub vegetation and/or associated fungi. Actinomycetota, Pseudomonadota, and Acidobacteriota were reported as the dominant taxa also in metagenomes and metatranscriptomes of tundra soil sampled from the same area (Pessi et al. 2022, Viitamäki et al. 2022). Our earlier studies of the same tundra habitats indicated a high dominance of Acidobacteriota in clone libraries of both WS and SA tundra heaths (Männistö et al. 2013). In this study, the dominance of Acidobacteriota was lesser with a greater abundance of Actinomycetota-associated reads. This increase in the share of Actinomycetota is likely due to differences in the DNA and RNA extraction protocol with stronger beat beating conditions used in our current protocol (described in Männistö et al. 2016). The most abundant bacterial OTUs were associated with the Actinomycetota genus *Acidothermus* which comprised ca. 20% of all reads and was especially dominant in the RNA-derived bacterial community both in WS and SA tundra heaths. *Acidothermus* spp. was recently reported as one of the most dominant genus-level taxa also in metagenomes and transcriptomes of soils sampled from the same area (Viitamäki et al. 2022). They were especially abundant in acidic shrub-dominated heaths, indicating that this taxon may have an important role in the organic-rich, nutrient-poor tundra soils. Members of Acidothermaceae were abundant also in alpine soils, where they increased with expansion of ericaceous shrubs (Broadbent et al. 2022) further indicating their association with ericaceous vegetation. The only described species of this genus is a thermophilic cellulose degrader (Mohagheghi et al. 1986) indicating that the role of tundra soil *Acidothermus* spp. may be associated with the decomposition of the large plant-derived organic stocks. As *Acidothermus* spp. belong to the order Frankiales, which contain well-known nitrogen-fixing species (Gtari et al. 2012) that may rise the question of whether the high abundance of these Actinomycetota is connected also to the nitrogen limitations of the habitat.

The most abundant class-level taxa in both WS and SA tundra soils were members of Alphaproteobacteria. The abundance and role of many of these alphaproteobacterial taxa may be associated with nitrogen acquisition as they are associated with known nitrogen-fixing genera (Tsoy et al. 2016). *Bradyrhizobium* and other Alphaproteobacteria were found to be dominant members of the denitrifier populations in the tundra soils of Kilpisjärvi where they were reported to encode terminal oxidases that are active both under highly aerobic conditions and those with high oxygen affinity (Pessi et al. 2022). This type of adaptation would give a competitive advantage for growth under waterlogged (spring) and dry conditions. *Bradyrhizobium* and many other alphaproteobacterial taxa have been also reported as dominant lignin-decomposing taxa in Alaskan tundra soils (Tao et al. 2020), indicating that in addition to a putative role in nitrogen cycling their abundance may be explained by their role in the decomposition of recalcitrant OM in organic-rich soils. The relatively small differences between bacterial communities in WS and SA tundra heaths may be due to the versatility of the dominant bacterial groups, with putative roles both in decomposition and nutrient acquisition. However, additional studies are needed to understand the mechanisms associated with these communities.

Although differences in vegetation and its mycorrhizal associations were likely important determinants for the soil microbial community composition, soil physico-chemical properties contributed significantly to both the bacterial and fungal community structure. Based on the DistLM model, the analyzed soil variables (pH, OM, and different N and P forms) explained roughly 20% and 26% of the variation in bacterial and fungal community structures, respectively. Apart from nutrient availability, pH and SOM were key determinants of active bacterial and fungal community structure. Of the analyzed soil properties, particularly soil pH has been shown to control the bacterial community structure in the same area (Männistö et al. 2007) as well as bacterial and fungal community structure globally (Lauber et al. 2009, Tedersoo et al. 2014). Although there were no significant differences is soil pH between the WS and SA sites, and the variation among different sample replicates was small, soil pH contributed significantly to the variation of bacterial and fungal community structures also in this study. However, as the soil pH was below 5 in all sites, the bacterial and fungal communities were dominated by acid-tolerant and oligotrophic taxa, which may constitute an important factor behind the similarities of the communities between WS and SA tundra heaths.

## Conclusions

As hypothesized, we found clear differences in the soil microbial communities between WS and SA tundra heaths. Snow-cover related to topography may, thus be an important microclimatic driver of both microbial community composition and soil C and N dynamics. WS heaths experienced very low wintertime soil temperatures and strong seasonal fluctuations, which drastically differed from the more stable soil temperature regimes of the SA heaths, but contrary to prediction, the soil microbial communities differed the most during the late growing season rather than during winter. Further contrasting predictions, we did not detect distinct cryotolerant communities in WS heaths during winter. A higher abundance of fungal PLFAs and a lower nutrient availability in the WS heaths together with the differing fungal community composition between the WS and SA heaths suggested that these communities were represented by stress-tolerant organisms adapted to nutrient-poor and cold soils. Previous studies have indicated that these types of microbial communities may be rather insensitive to extreme temperatures (Männistö et al. 2018). Instead, the seasonal patterns in microbial biomass and community composition closely followed the seasonal patterns in soil nutrient availability, especially in the more nutrient-limited WS tundra heaths. These results suggest that, rather than directly through low wintertime soil temperatures, topographic differences shape soil microbial communities through modifying the dominant vegetation and soil nutrient availability. This could partially explain why different snow-manipulation experiments have found inconsistent effects of snowpack on microbial community structure and function, ranging from no effect to transient or legacy effects on bacterial and fungal communities in tundra, forest, and alpine soils (Aanderud et al. 2013, Morgado et al. 2016, Mundra et al. 2016, Ricketts et al. 2016, Gavazov et al. 2017, Männistö et al. 2018, Voříšková et al. 2019). Future experimental studies on Arctic soils should, thus include several habitat types with divergent dominant vegetation and nutrient levels to fully separate the direct and indirect effects of temperature.

In the future, the ongoing climate warming will lead to more frequent freeze–thaw cycles and a lower duration and insulation of the snow cover (Bintanja and Andry 2017, McCrystall et al. 2021, Serreze et al. 2021). According to our findings from current topographic gradients in snow accumulation, owing to the high stress-tolerance of soil microbial communities in acidic soils with low temperatures (Männistö et al. 2018), these climatic changes may potentially have a minor role for future soil microbial communities. Instead, shifts in microbial community composition in response to climate warming will likely be largely mediated by shifts in the dominant vegetation and corresponding effects on mycorrhizal associations as well as substrate and nutrient availability for soil microorganisms. Assuming that the ongoing expansion of deciduous shrubs continues across the circumpolar Arctic (e.g. Myers-Smith et al. 2020), counterintuitively, soil microbial communities similar to what we detected for SA tundra heaths might, therefore increase in coverage.

## Supplementary Material

fiae036_Supplemental_Files
