# Supplementary material for: Bacterial and fungal communities in sub-Arctic tundra heaths are shaped by contrasting snow accumulation and nutrient availability
Source: FEMS Microbiol Ecol. 2024 Mar 28;100(4):fiae036. doi: 10.1093/femsec/fiae036 (PMC10996926; doi:10.1093/femsec/fiae036)
Supplement: fiae036_Supplemental_Files [file fiae036_supplemental_files.zip › supp data Fig_S2-S3.docx]

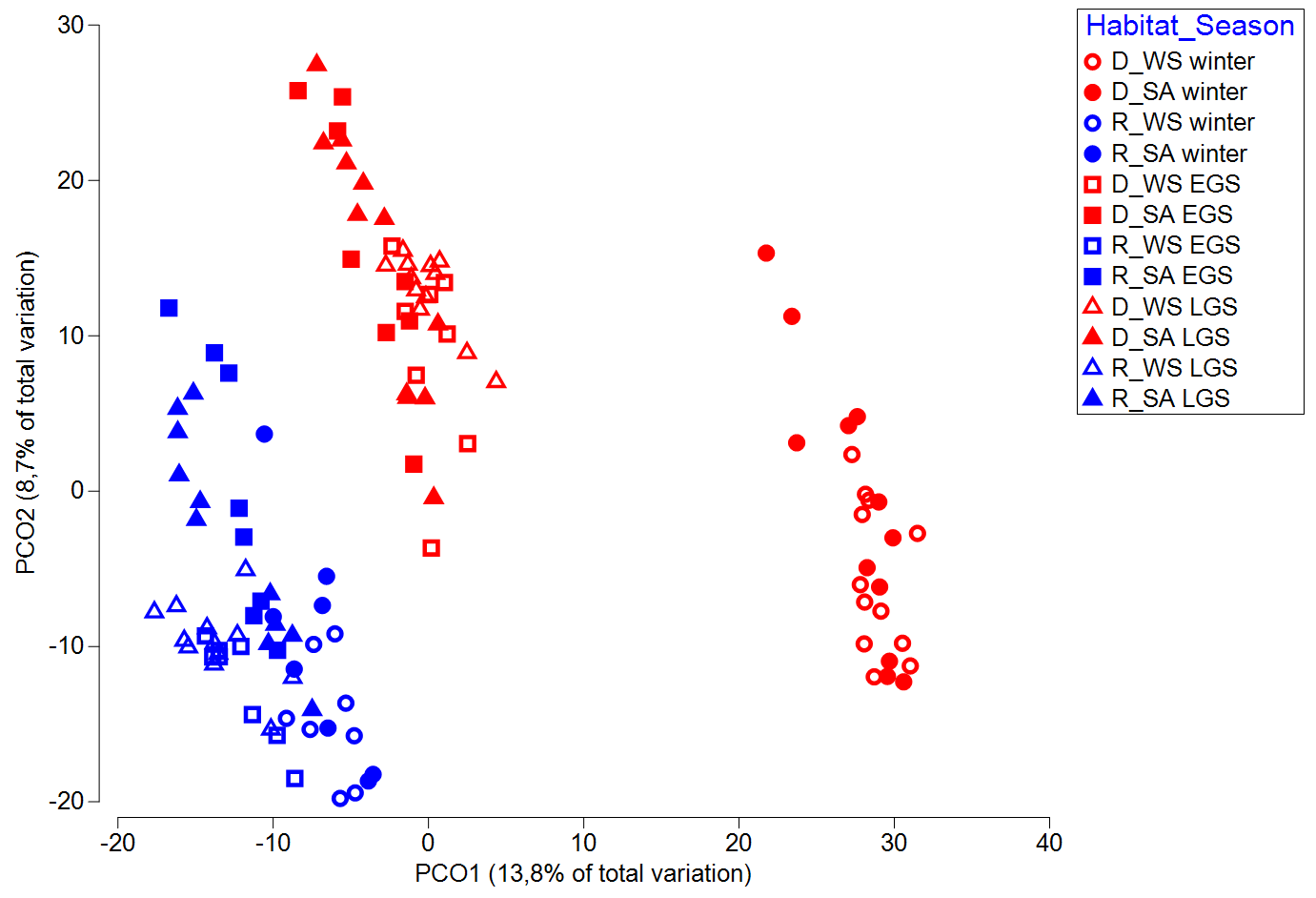


DNA_winter

Supplementary Figure S2. PCO ordination of active (RNA derived) and total (DNA derived) bacterial communities in WS and SA tundra heaths sampled in winter, early and late growing season showing the separation of the winter DNA derived community from all other sampling seasons. Abbreviations: D=DNA, R=RNA, WS=windswept, SA=snow accumulating, EGS=early growing season, LGS=late growing season.


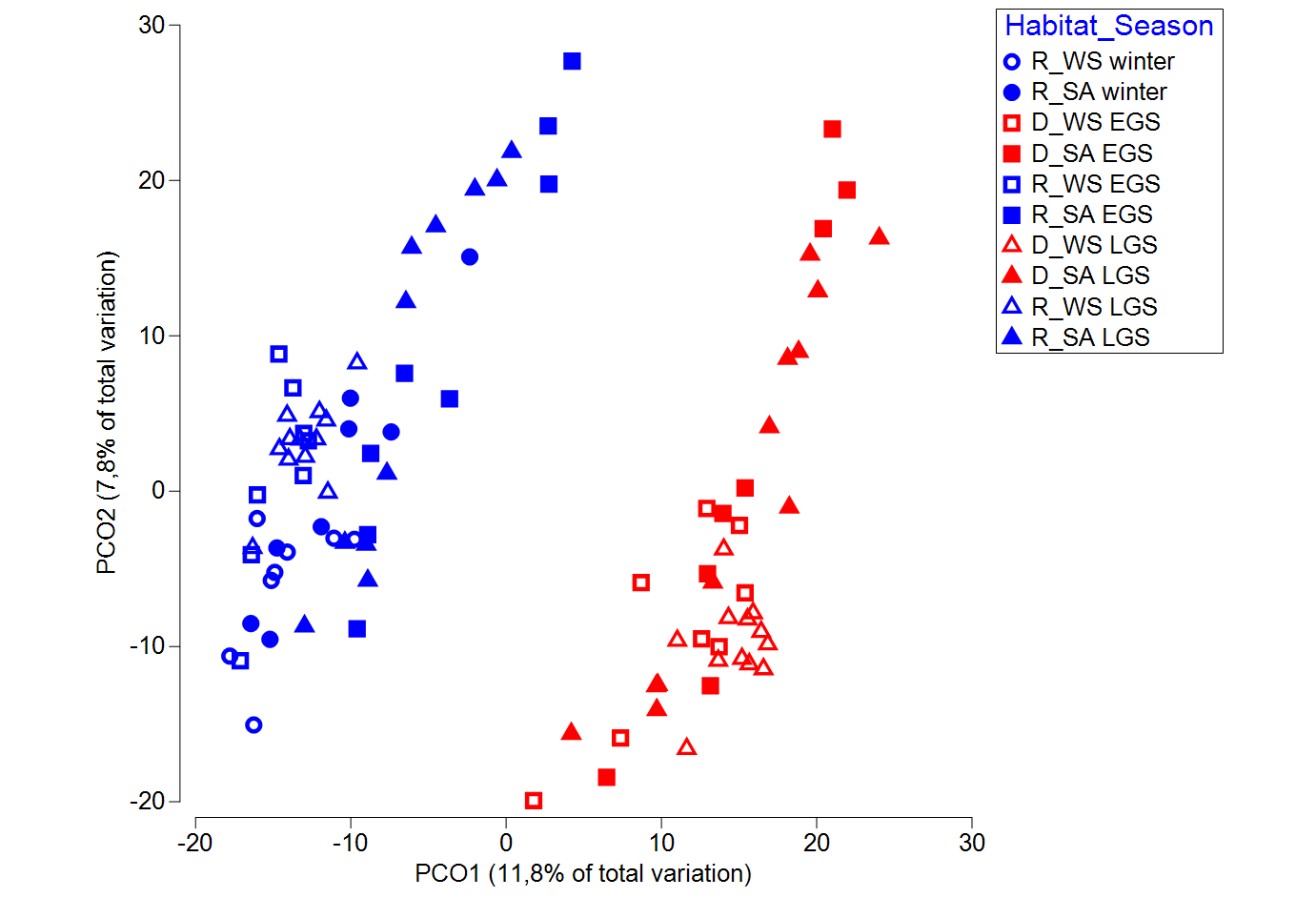

Supplementary Figure S3. PCO ordination and relative abundance of bacterial genera in the DNA and RNA derived bacterial communities of WS and SA tundra heaths. Winter DNA samples are excluded from the PCO ordination. Abbreviations: D=DNA, R=RNA, WS=windswept, SA=snow accumulating, EGS=early growing season, LGS=late growing season.
