# Supplementary material for: Bacterial and fungal communities in sub-Arctic tundra heaths are shaped by contrasting snow accumulation and nutrient availability
Source: FEMS Microbiol Ecol. 2024 Mar 28;100(4):fiae036. doi: 10.1093/femsec/fiae036 (PMC10996926; doi:10.1093/femsec/fiae036)
Supplement: fiae036_Supplemental_Files [file fiae036_supplemental_files.zip › supp data Fig_S4_S5.docx]

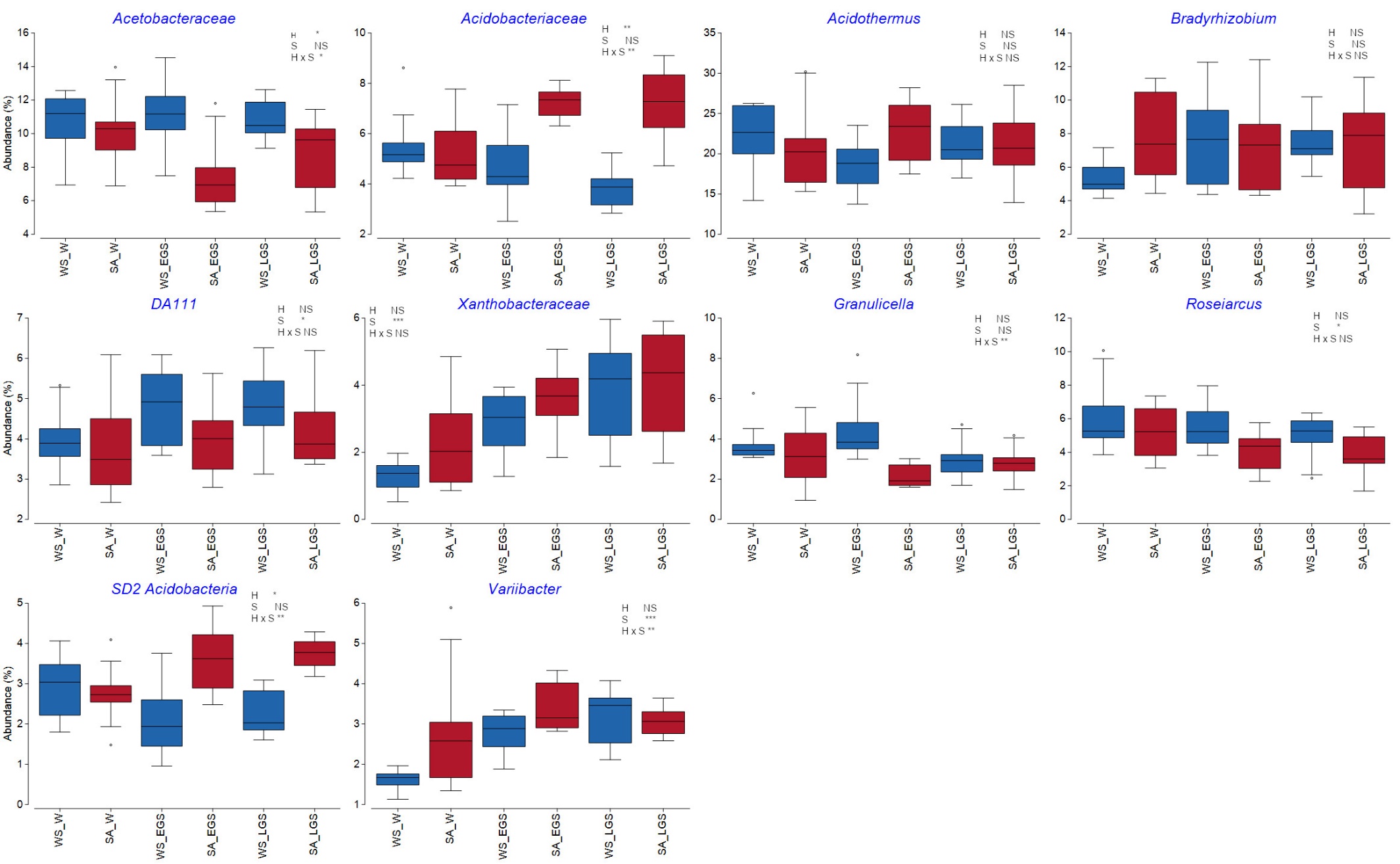


Figure S4. Abundance of the ten most dominating bacterial genera in RNA samples from windswept and snow-accumulating tundra heaths sampled in winter (Feb), early growing season (June) or late growing season (Sept). Significant effects of habitat, season or their interactions were tested using PERMANOVA. Significance levels ***, p<0.001; **, p<0.01; *, p<0.05; NS, not significant


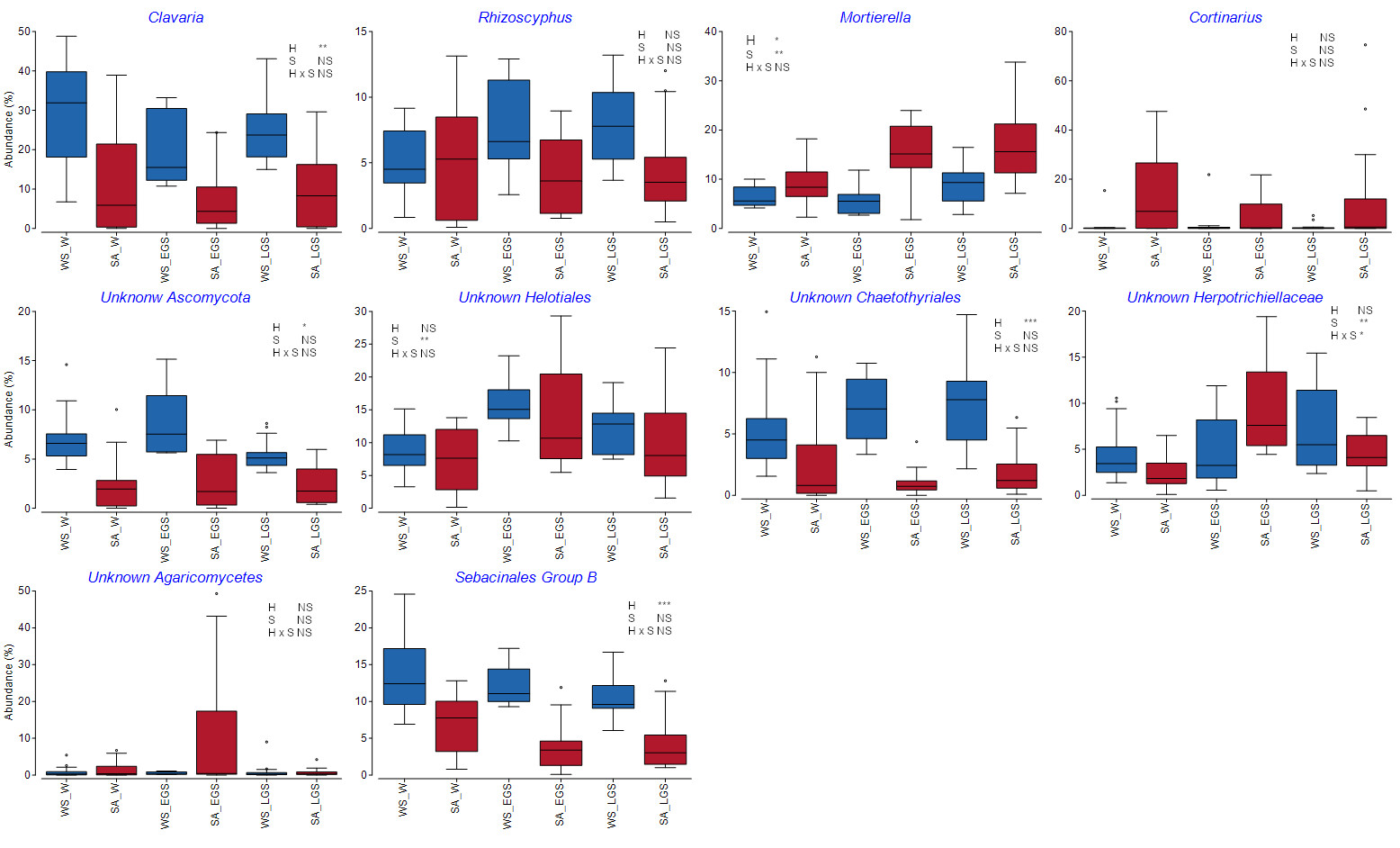


Figure S5. Abundance of the ten most dominating fungal genera in RNA samples from windswept and snow-accumulating tundra heaths sampled in winter (Feb), early growing season (June) or late growing season (Sept). Significant effects of habitat, season or their interactions were tested using PERMANOVA. Significance levels ***, p<0.001; **, p<0.01; *, p<0.05; NS, not significant
