# Supplementary material for: Bacterial and fungal communities in sub-Arctic tundra heaths are shaped by contrasting snow accumulation and nutrient availability
Source: FEMS Microbiol Ecol. 2024 Mar 28;100(4):fiae036. doi: 10.1093/femsec/fiae036 (PMC10996926; doi:10.1093/femsec/fiae036)
Supplement: fiae036_Supplemental_Files [file fiae036_supplemental_files.zip › Supplementary data Figures legends.docx]

Supplementary Figures

Figure S1. Pictures of the field site: North side of Mt Pikku-Malla showing windswept ridges and snow accumulating depressions (A), sampling of deep snow (B) and windswept (C) plots, vegetation of snow accumulating (D) and windswept (E) plots, field site in winter (F) and summer (G)

Figure S2. PCO ordination of active (RNA derived) and total (DNA derived) bacterial communities in WS and SA tundra heaths sampled in winter, early and late growing season showing the separation of the winter DNA derived community from all other sampling seasons. Abbreviations: D=DNA, R=RNA, WS=windswept, SA=snow accumulating, EGS=early growing season, LGS=late growing season.

Figure S3. PCO ordination and relative abundance of bacterial genera in the DNA and RNA derived bacterial communities of WS and SA tundra heaths. Winter DNA samples are excluded from the PCO ordination. Abbreviations: D=DNA, R=RNA, WS=windswept, SA=snow accumulating, EGS=early growing season, LGS=late growing season.

Figure S4. Abundance of the ten most dominating bacterial genera in RNA samples from windswept and snow-accumulating tundra heaths sampled in winter (Feb), early growing season (June) or late growing season (Sept). Significant effects of habitat, season or their interactions were tested using PERMANOVA. Significance levels ***, p<0.001; **, p<0.01; *, p<0.05; NS, not significant.

Figure S5. Abundance of the ten most dominating fungal genera in RNA samples from windswept and snow-accumulating tundra heaths sampled in winter (Feb), early growing season (June) or late growing season (Sept). Significant effects of habitat, season or their interactions were tested using PERMANOVA. Significance levels ***, p<0.001; **, p<0.01; *, p<0.05; NS, not significant.

Figure S6. Figure S6. Distance based redundancy analysis plots showing the best predictors in the DistLM model explaining active bacterial (A), total bacterial (B) and fungal (C) community structure.
