# Supplementary material for: Bacterial and fungal communities in sub-Arctic tundra heaths are shaped by contrasting snow accumulation and nutrient availability
Source: FEMS Microbiol Ecol. 2024 Mar 28;100(4):fiae036. doi: 10.1093/femsec/fiae036 (PMC10996926; doi:10.1093/femsec/fiae036)
Supplement: fiae036_Supplemental_Files [file fiae036_supplemental_files.zip › Supplementary data Tables.docx]

**Table S1**. Effect of habitat (WS or SA), sampling season (winter, early and late GS) and their interaction on soil physico-chemical properties and microbial biomass proxies. F and p values were obtained by linear mixed effect model with habitat and season as fixed factors and site as a random factor. Season was assigned as a repeated factor. Logaritmic transformations (indicates as *) were used to meet the assumptions of linear mixed model.

|  |  | Habitat |  | Season |  | Habitat x Season | |
| --- | --- | --- | --- | --- | --- | --- | --- |
|  |  | *F* | *p* | *F* | *p* | *F* | *p* |
| pH |  | 2.496 | 0.168 | 1.364 | 0.265 | 0.185 | 0.832 |
| OM (%) |  | 0.310 | 0.598 | 0.011 | 0.989 | 0.078 | 0.925 |
| DW (%) |  | 0.035 | 0.857 | 2.224 | 0.122 | 1.245 | 0.299 |
| N_tot_ (mgkg^-1^)* |  | 21.340 | **0.004** | 15.896 | **<0.001** | 3.810 | 0.031 |
| N_org_ (mgkg^-1^) |  | 17.053 | **0.007** | 4.797 | 0.012 | 0.529 | 0.592 |
| NO_3_ (mgkg^-1^) |  | 3.313 | 0.116 | 6.248 | **0.004** | 6.030 | **0.004** |
| NH_4_ (mgkg^-1^)* |  | 7.534 | 0.034 | 5.571 | **0.007** | 1.912 | 0.161 |
| N_mic_ (mgkg^-1^) |  | 5.353 | 0.060 | 15.032 | **<0.001** | 0.050 | 0.951 |
| P_tot_ (mgkg^-1^)* |  | 10.675 | 0.017 | 4.622 | 0.015 | 0.279 | 0.758 |
| P_mic_(mgkg^-1^)* |  | 0.011 | 0.922 | 1.918 | 0.159 | 1.895 | 0.162 |
| Total PLFA |  | 0.018 | 0.897 | 1.618 | 0.220 | 0.309 | 0.737 |
| Bacterial PLFA |  | 1.567 | 0.259 | 1.151 | 0.333 | 0.262 | 0.771 |
| Fungal PLFA |  | 16.208 | **0.007** | 2.732 | 0.086 | 0.233 | 0.794 |
| F/B ratio |  | 82.804 | **<0.001** | 4.728 | 0.019 | 1.109 | 0.347 |
| 16S copies* |  | **31.165** | **0.001** | 38.792 | **<0.001** | 54.388 | **<0.001** |
| ITS copies* |  | 0.472 | 0.517 | 13.685 | **<0.001** | 9.729 | **<0.001** |

Significance levels: p<0.05 are underlined, p<0.01 are indicated by bold

**Table S2.** Marginal tests for DistLM analysis showing the contribution of the tested soil parameters on active (RNA) bacterial, total (DNA) bacterial and fungal comm8unity structure. Marginal test indicates the proportion of variation that is explained by the individual factors without considering the other factors. SS, sum of squares; F, F distribution; P, P value; Prop., the proportion of explained variation. Significance levels: p<0.05 are underlined, p<0.01 are indicated by bold

**Bacteria RNA**

| Variable | SS(trace) | Pseudo-F | P | Prop. |
| --- | --- | --- | --- | --- |
| NO_3_ | 1600,5 | 1,195 | 0,12 | 0,022051 |
| **NH_4_^*^** | 3124,6 | 2,3843 | **0,001** | 0,04305 |
| **N_tot_^*^** | 4199,2 | 3,2546 | **0,001** | 0,057855 |
| **N_mic_** | 3600,3 | 2,7662 | **0,001** | 0,049604 |
| **P_tot_^*^** | 4675,4 | 3,6491 | **0,001** | 0,064416 |
| **P_mic_^*^** | 5079,2 | 3,988 | **0,001** | 0,069979 |
| **OM** | 2986,4 | 2,2743 | **0,001** | 0,041146 |
| **pH** | 4367,5 | 3,3934 | **0,001** | 0,060174 |
| **DW** | 2259,6 | 1,703 | **0,004** | 0,031132 |

**Bacteria DNA**

| Variable | SS(trace) | Pseudo-F | P | Prop. |
| --- | --- | --- | --- | --- |
| NO_3_ | 1200,4 | 0,89444 | 0,698 | 0,022997 |
| **NH_4_^*^** | 3591,6 | 2,8078 | **0,001** | 0,068805 |
| **N_tot_^*^** | 4000,9 | 3,1543 | **0,001** | 0,076646 |
| **N_mic_** | 3219,8 | 2,498 | **0,001** | 0,061681 |
| **P_tot_^*^** | 3548,6 | 2,7717 | **0,001** | 0,067981 |
| **P_mic_^*^** | 2963,9 | 2,2875 | **0,001** | 0,05678 |
| **OM** | 2443,2 | 1,8659 | **0,004** | 0,046805 |
| **pH** | 3552,8 | 2,7752 | **0,001** | 0,068061 |
| DW | 1938,9 | 1,4659 | 0,033 | 0,037144 |

**Fungi DNA**

| Variable | SS(trace) | Pseudo-F | P | Prop. |
| --- | --- | --- | --- | --- |
| NO_3_ | 3120 | 1,2708 | 0,14 | 0,020408 |
| **NH_4_^*^** | 14519 | 6,4009 | **0,001** | 0,094968 |
| **N_tot_^*^** | 20461 | 9,4254 | **0,001** | 0,13384 |
| **N_mic_** | 9620,3 | 4,0963 | **0,001** | 0,062927 |
| **P_tot_^*^** | 15788 | 7,0248 | **0,001** | 0,10327 |
| **P_mic_^*^** | 7134,3 | 2,986 | **0,001** | 0,046666 |
| **OM** | 6206,7 | 2,5813 | **0,002** | 0,040599 |
| **pH** | 9375,8 | 3,9854 | **0,001** | 0,061328 |
| **DW** | 4972,1 | 2,0506 | **0,009** | 0,032523 |
