# Supplementary figures and images for: Bacterial and fungal communities in sub-Arctic tundra heaths are shaped by contrasting snow accumulation and nutrient availability
Source: FEMS Microbiol Ecol. 2024 Mar 28;100(4):fiae036. doi: 10.1093/femsec/fiae036 (PMC10996926; doi:10.1093/femsec/fiae036)

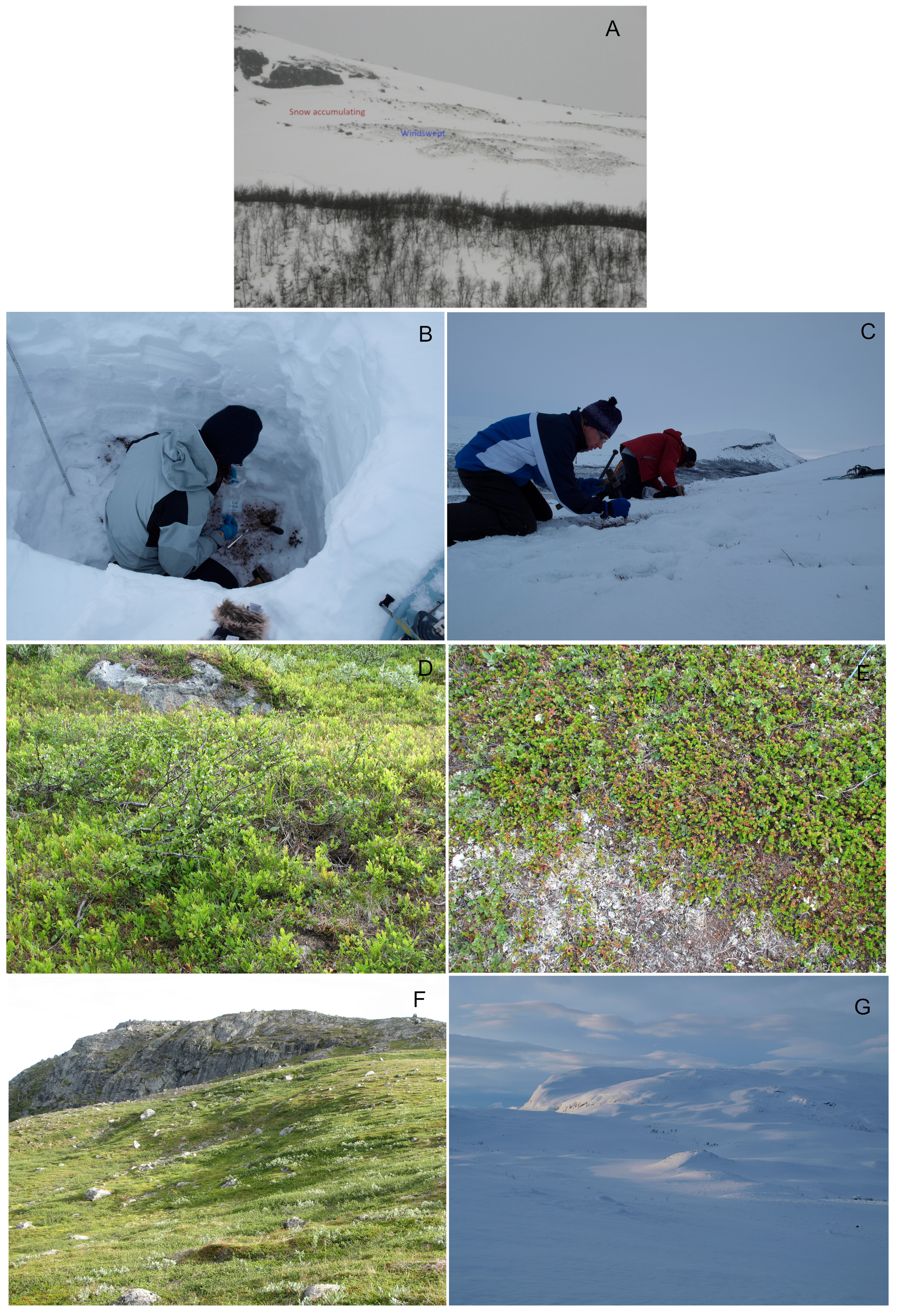

Supplement: fiae036_Supplemental_Files [file fiae036_supplemental_files.zip › supp data Fig_S1.jpg]
